# A practice facilitation-guided intervention in primary care settings to reduce cardiovascular disease risk: a cost analysis

**DOI:** 10.1186/s43058-021-00116-x

**Published:** 2021-02-06

**Authors:** Heather T. Gold, Nina Siman, Allison M. Cuthel, Ann M. Nguyen, Hang Pham-Singer, Carolyn A. Berry, Donna R. Shelley

**Affiliations:** 1 Department of Population Health, NYU Langone Health, New York, NY USA; 2 Ronald O. Perelman Department of Emergency Medicine, NYU Langone Health, New York, NY USA; 3 Rutgers Center for State Health Policy, Rutgers University, New Brunswick, NJ USA; 4 New York City Department of Health and Mental Hygiene, New York, NY USA; 5 Department of Policy and Public Health Management, School of Global Public Health, New York University, New York, NY USA

**Keywords:** Practice facilitation, Cost analysis, Clinical guideline adherence, Cardiovascular disease prevention

## Abstract

**Background:**

A stepped-wedge, cluster randomized controlled trial assessed the effectiveness of practice facilitation (PF) for adoption of guidelines for prevention and treatment of cardiovascular disease risk factors. This study estimated the associated cost of PF for guideline adoption in small, private primary care practices.

**Methods:**

The cost analysis included categories for start-up costs, intervention costs, and practice staff costs for the implemented PF-guided intervention. We estimated the total 1-year costs to operate the program and calculated the mean and range of the cost-per-practice by quarter of the intervention. We estimated the lower and upper bounds for all salary expenses, rounding to the nearest $100.

**Results:**

Total 1-year intervention costs for all 261 practices ranged from $7,900,000 to $10,200,000, with program and practice salaries comprising $6,600,000–$8,400,000 of the total. Start-up costs were a small proportion (3%) of the total 1-year costs. Excluding start-up costs, quarter 1 cost-per-practice was the most expensive at $20,400–$26,700, and quarter 4 was the least expensive at about $10,000. Practice staff time (compared with program staff time) was the majority of the staffing costs at 75–84%.

**Conclusions:**

The PF strategy costs approximately $10,000 per practice per quarter for program and practice costs, once implemented and running at highest efficiency. Whether this program is “worth it” to the decision-maker depends on the relative costs and effectiveness of their other options for improving cardiovascular risk reduction.

**Trial registration:**

This study is retrospectively registered on January 5, 2016, at www.clinicaltrials.gov as NCT02646488.

Contributions to the literature

- Practice facilitation was used as an implementation strategy to help clinical practices adhere more closely to primary care practice guidelines, yet prior to this, few studies have estimated the cost of this strategy, and none is comparable in approach.
- The intervention and its implementation cost about $10,000 per urban primary care practice once the program was in place and effective for 9 months.
- Understanding the effectiveness and cost trade-offs of any implementation strategy and evidence-based intervention can aid decision-makers in their effort to adopt cost-effective approaches to improving adherence to clinical practice guidelines; here we showed that practice facilitation as implemented was likely not affordable for small primary care practices.

## Background

Clinical guidelines for reducing cardiovascular disease (CVD) risk factors are not consistently integrated into primary care practice [1, 2]. One approach to increasing adoption of clinical guidelines is practice facilitation (PF) [3–11]. PF is an implementation strategy that emphasizes building practice and organizational capacity to implement innovations in health care delivery into routine primary care practice [5]. Several studies have found that PF is effective in improving adoption of a range of preventive care guidelines, but this research largely has focused on implementing guidelines for single risk factors rather than addressing the complexity of simultaneous guideline implementation across multiple risk factors.

HealthyHearts New York City (HHNYC) was one of seven cooperatives funded through the Agency for Healthcare Research and Quality’s national EvidenceNOW initiative to reduce CVD risk. The study was designed to assess the effect of practice facilitation on adoption of Million Hearts ABCS outcomes defined as (A) aspirin when indicated, (B) blood pressure control, (C) cholesterol management, and (S) smoking screening and cessation intervention [3]. The main risk factor analysis found a significant effect of PF on the smoking composite measure and the proportion of smokers counseled only [12]. The PF literature demonstrates the effectiveness of PF, but most of those studies aimed to improve a single measure (e.g., cervical cancer screening rates or hypertension management). A longer intervention might be necessary to show effectiveness because of the added complexity when supporting system changes to improve a range of prevention and treatment risk factors, which requires changing many care processes at once to achieve guideline-recommended care [13].

The process assessment found a high level of fidelity as measured across four categories: frequency, duration, content, and coverage [14]. All practices received at least 10 of the planned 13 visits by a PF, and over 94% received 13 or more visits. Over 95% of practices received all planned content (introductory and ABCS-specific tasks), and facilitators achieved just over 65% coverage, that is, achieving every goal comprising the number of visits (13), tasks (39), and education on strategies (27). There were no meaningful differences in practice characteristics between those that achieved or did not achieve high rates of fidelity for this complex intervention [14].

Although there is growing interest in understanding how PF can effectively improve patient care and health care outcomes across a multitude of care processes, there is a lack of data on the cost of this strategy. To the best of our knowledge, only two articles have reported on the cost of PF interventions [15, 16], each using a different cost-analysis approach, with one from the USA (2013) and an older one ending in 1998 from Canada (2005). The gaps in the literature are due to the limited cost analyses overall and the variation in interventions and analyses that make comparability challenging. One of these studies was based in South Texas, focused on diabetes only with a much lower intensity (6 PF visits in 12 months), and excluded start-up costs but included travel and food costs [16]; the other was based in Ontario, Canada, and was high intensity (33 visits over 18 months) and included intervention, treatment, and follow-up costs for preventive services used or avoided and essentially was a cost-consequence modeling study incorporating total estimated intervention costs, but the analysis was based on many assumptions beyond data collected directly in the study.

Various stakeholders interested in advancing the use of PF to optimize adoption of clinical guidelines (e.g., policymakers, practice-based research networks, independent practice associations) would benefit from having cost information and analysis [17]. This article aimed to fill gaps in the literature by estimating the start-up costs, salary expenses, and cost-per-practice for the implemented PF-guided intervention.

## Methods

### Study setting and design

HealthyHearts New York City (HHNYC) is a partnership between the New York University School of Medicine; and the Primary Care Information Project (PCIP), a bureau of the New York City Department of Health and Mental Hygiene. The study was a stepped-wedge, cluster randomized controlled design in which sites were randomized into one of four 12-month intervention waves to evaluate the effect of practice facilitation (implementation strategy) to help small independent practices adopt CVD guidelines (intervention). A new wave of practices was enrolled every 3 months. The protocol for the PF implementation strategy consisted of a minimum of 13 in-person, on-site PF visits to an assigned panel of practices within a 1-year intervention period to assist practices with their implementation of system changes consistent with the patient-centered medical home and the Chronic Care Model (CCM) [18, 19]. For example, in alignment with the CCM’s “delivery system redesign” component, facilitators promoted the use of patient registries to identify high-risk patients. PFs scheduled two visits to their assigned practices in the first month and monthly visits for the remaining 11 months. For the cost analysis, we included the 261 small independent practices from the PCIP network that were enrolled during the first year of the study intervention phase (1 December 2015–30 November 2016). Sixteen practice facilitators were assigned to work across the practices.

Nearly two-thirds of the 261 practices were solo clinicians (*n*=149), 37% were in accountable care organizations [20], almost half (47%) were in medically underserved areas [21] and/or designated as patient-centered medical homes, and 45% of patients had Medicaid.

#### Data sources

There were several data sources for the cost analysis, comprising categories for start-up costs, implementation and intervention costs, and practice staff costs.

##### Start-up costs

The program manager tracked all start-up costs in a database that included materials, supplies, toolkits for the facilitators and for each practice to implement the CCM and evidence-based guidelines for the ABCS measures, computers for use by the facilitators, webinar hosting, and program staff time and expenses for kickoff events, training, and meetings prior to program launch, based on invoices from and communication with PCIP. Total start-up costs included all of the above-mentioned costs, but excluded time costs for practices, because while we documented the number of staff and clinicians present, we did not know which specific staff members attended.

##### Implementation and intervention costs

Facilitators used an electronic customer relationship management (CRM) system (i.e., Salesforce™) to document the amount of time spent at each visit and other interactions including email and phone calls. The facilitator costs also included other meetings related to the project that did not involve site contact, such as supervision and project management meetings with other facilitators and project or program staff. Facilitator travel time was incorporated as a regular part of their workday and not calculated separately. Further, research costs were not included, because they would not be part of a future implementation.

In addition to facilitators, we accounted for the time of facilitator managers and several other program staff (e.g., outreach coordinators). Data for salaries were obtained either from published salary ranges from the New York City Department of Health and Mental Hygiene or estimated from the 2016 data of the U.S. Bureau of Labor Statistics, where we assigned a dollar value for each hour of staff time based on the national wage and fringe rate of those who could perform the activity in the same setting. We estimated the staff time cost with a lower and upper bound based on the given New York City Department of Health and Mental Hygiene salary range (e.g., $74,700–85,700/year for a practice facilitator including fringe) or from possible occupation types from the U.S. Bureau of Labor Statistics. Several occupation types could fulfill the duties of different program staff, such as nurse practitioner ($49.54/hour including fringe), registered nurse ($31.68/hour including fringe), or medical assistant ($15.76/hour including fringe), and all have different wage levels [22].

##### Practice staff costs

The HHNYC practice member survey was used to estimate the amount of time physicians and their practice staff spent on activities that were implemented as part of the intervention. (Questions were essentially, “Does your practice site conduct [daily huddles *or* outreach to high-risk patients]?” with a skip pattern allowing for recall of number of minutes per most recent full day or week spent on the activity.) The surveys were completed by all providers and staff at 3 times (baseline, post-intervention, and 6-month follow-up). We used 2016 data from the U.S. Bureau of Labor Statistics to estimate hourly wages including fringe for practice staff time involved in the program [22]. Examples of personnel included medical biller, financial controller, front desk staff, general and operations manager, medical assistant, medical secretary, nurse practitioner, office manager, physician assistant, physician, registered nurse, and secretary/administrative assistant. We used the national average wage as the upper bound on time costs and 80% of that as a lower bound.

### Analytic approach

Our analyses distinguished between start-up costs (e.g., kickoff events, program staff time), program staff costs (i.e., facilitator and associated staff time), and practice costs (e.g., provider and practice staff) [23–25]. These categories allowed for estimating the perspectives of both the entity that must decide to pay for the initial program and of the practice. We estimated the total 1-year costs to operate the program and calculated the mean and range of the cost-per-practice by quarter of the intervention, knowing that facilitators increased their volume of practices as the year progressed (i.e., started at less than full capacity). That is, Wave 1, quarter 1, had the lowest number of practices per facilitator, and this increased through the year. By estimating the cost-per-practice, we indicate how program efficiency over time might change the cost-per-practice over 1-year duration, as facilitators operated at closer to full capacity. We estimated the lower and upper bounds for all salary expenses, rounding to the nearest $100.

## Results

Table 1 shows the estimated costs for the 1-year PF program encompassing 261 primary care practices. Total intervention cost estimates ranged from $7,900,000 to $10,200,000, with program and practice salaries comprising $6,600,000 to $8,400,000 of the total. Start-up costs for the PF program ranged from $311,500 to $315,000. Start-up costs were a small proportion (3%) of the total 1-year costs.

**Table 1:** Costs for practice facilitation program (2016 US$)

Cost category	Minimum ($)	Maximum ($)	Mean ($)
Total 1-year costs	7,933,300	10,163,300	9,048,300
1-year program staff salaries (wage+fringe)	1,546,700	2,318,300	1,932,500
1-year practice staff salaries (wage+fringe)	6,296,100	7,870,100	7,083,100
Start-up costs	311,500	315,000	313,300
Materials and supplies	299,400	299,400	299,400
Meetings and kickoff events staffing	6800	10,300	8600
Cost-per-practice, excluding start-up costs			
Q1	20,400	26,700	23,600
Q2	12,500	16,100	14,300
Q3	10,400	13,400	11,900
Q4	8700	11,200	10,000
Percentage of cost/practice comprising practice staff cost			
Q1	77%	74%	75%
Q2	81%	79%	80%
Q3	84%	82%	83%
Q4	85%	83%	84%
Total start-up costs/#practices	1200	1200	1200

*Note*: Ranges based on salary ranges, using minimum and maximum of given ranges from the New York City Department of Health and Mental Hygiene or by occupation type from the U.S. Bureau of Labor Statistics. Rounded to nearest $100

The number of practices participating increased over the year, as planned, (Table 2) yielding a varying staff cost-per-practice by quarter. Excluding start-up costs, quarter 1 was the most expensive at $20,400–$26,700 per practice, followed by a decreasing quarterly cost-per-practice over time, averaging $14,300, $11,900, and $10,000 in quarters 2–4, respectively. Practice staff time (compared with program staff time) was the majority of the staffing costs at 75–84%, depending on the quarter (Table 1).

**Table 2:** Number of enrolled practices, by quarter

Quarter of year 1 rollout	Number of enrolled practices
Q1	70
Q2	143
Q3	205
Q4	261

## Discussion

Our findings showed that once scaled-up, the cost-per-practice of a complex PF intervention conducted in small urban primary care practices was as low as $10,000 (2016US$) for the program and practice staff time. However, policymakers and health care system leaders will not be inclined to pay for a PF program if it is not achieving stated goals. Despite high levels of fidelity to the intensive PF protocol, operationalizing the large number of changes required to concurrently improve guideline adoption across a broad range of chronic disease indicators was challenging. We have documented several potential explanations for the lack of change in the other three risk factor measures, including that some clinics began the study with relatively high rates of meeting targets. Because intervening on multiple, rather than single, risk factor targets and measures is more complex and less studied, it was clear that the PFs may have needed more time to partner with practices to implement capacity-building activities and the necessary system changes, which would increase costs.

In addition, basing an assessment of the value of PF solely on short-term changes in clinical risk factor measures may underestimate the value of PF. Qualitative interviews with practices enrolled in HHNYC found that small independent practices viewed facilitation as an important resource, particularly because they offered support to the practices that was unavailable onsite to optimize their use of the EHR for identifying and addressing other quality improvement gaps.

Only two articles have reported on the cost of PF-guided interventions [15, 16]. A Canada-based study of a PF-guided intervention to improve preventive services estimated a cost of $10,835 (1999 CAD$) per practice [15]. Culler reported on a PF-guided intervention to improve diabetes care, with an estimated median cost of $9670 (US$, data year unknown) per practice, depending on wages and the intensity of the intervention [16]. The two studies had different ways of measuring costs, different target populations, and different intervention components; however, their findings were relatively similar to one another and to ours. We encourage others to report on programmatic costs, consequences, and components to help build a greater understanding of PF program costs.

This study had limitations. Given the setting of our study, our estimates should be considered conservative, as PCIP provided an existing infrastructure for the intervention, eliminating costs such as practice recruitment and extensive PF training. If facilitators are unavailable at the time of program initiation and new ones need to be recruited and/or trained to be general facilitators (i.e., separate from intervention-specific training), the program cost may be underestimated [26]. Because travel costs were included as part of a facilitator’s day, but not calculated separately, we may be overestimating the cost of the PF program if implemented elsewhere; in a smaller geographic area, travel time could be much lower than that required for public transportation in New York City, and therefore, the facilitator could meet with more practices per day and be more efficient. The more practices that a facilitator manages, the lower the total cost-per-practice (i.e., increase the denominator); on the other hand, the practice-specific costs may not change. In addition, we did not capture practice-based remuneration for activities like smoking cessation counseling, which is billable. Also, note that we included the cost of report generation as a program cost in this study because they were created by the facilitators, but they typically would be a practice-associated cost. As noted earlier, total start-up costs excluded time costs for practices, because while we documented the number of staff and clinicians present, we did not know which specific staff members attended; this would lead to an underestimate of total costs. Finally, this program involved working with an organization that is part of a large, urban health department, which has been doing similar work in the past and had some pre-existing infrastructure in place prior to implementation (e.g., staff activity tracking via Salesforce software).

This program reached up to 261 practices by its final quarter at a total mean cost of $9 million. In order for any health care organization or individual practice to decide whether this PF-guided program is reasonable to implement, they need to know the effectiveness of such a program first, followed by an understanding of total costs from their perspective, that is, to their practice. Based on the relatively high quarterly costs, from a maximum of $19,600 early in the program to a minimum of $7400 in the final quarter, small independent practices could not afford a PF service. However, this emphasizes the role of organizations like PCIP that have the infrastructure and mission to support practice improvement by offering shared resources like practice facilitation. However, PCIP and other stakeholders who provide PF services need to be able to assess the cost of this service. Further, this study’s PF protocol required training and monthly meetings that generated a cost ranging from $16/h (medical assistant) to $50/h (nurse practitioner) or more for physicians, plus fringe, depending on which practice staff worked with the facilitator [22]. If lower-paid staff could substitute in the practice, costs might be lower. This analysis showed total costs broken into relevant categories for different stakeholder perspectives, which can be useful for decision-making by these parties.

## Conclusion

In conclusion, the HHNYC PF program costs approximately $10,000 per practice per quarter for program and practice costs, once implemented and running at the highest efficiency. Start-up costs were a small portion of total overall costs, however, and earlier-quarter estimates of cost-per-practice were high. Whether this program is “worth it” to the decision-maker depends on the relative costs and effectiveness of their other options for improving the ABCS outcomes of (A) appropriate aspirin use, (B) blood pressure control, (C) cholesterol management, and (S) smoking cessation.

## Data Availability

The datasets used and/or analyzed during the current study are available from the corresponding author on reasonable request.
